# Making way for a clinical feedback system in the narrow space between sessions: navigating competing demands in complex healthcare settings

**DOI:** 10.1186/s13033-019-0324-5

**Published:** 2019-11-02

**Authors:** Runar Tengel Hovland, Christian Moltu

**Affiliations:** 1 Department of Health and Caring Sciences, Western Norway University of Applied Sciences, Førde, Norway; 2 Department of Psychiatry, District General Hospital of Førde, Førde, Norway

**Keywords:** Implementation research, Clinical feedback systems, Norse feedback, Complex adaptive systems

## Abstract

**Background:**

Although substantial empirical research supports the clinical value of routine outcome measures/clinical feedback systems (ROM/CFS), translation into routine practice poses several challenges. The present case study investigated how stakeholders, clinicians, patients and clinical managers related to the implementation of the Norse Feedback (NF) in ordinary practice.

**Methods:**

We did an in-depth qualitative case study of the implementation of NF in a public mental-health institution. The settings were two outpatient clinics and two in-patient clinics organized under the same health trust. Data were drawn from three sources: archival sources (n = 16), field notes (n = 23), and 43 in-depth interviews with clinicians (n = 19), clinical managers (n = 5) and patients (n = 12). Ten of the participants were interviewed twice. The data were coded inductively and analyzed using a stringent qualitative methodology.

**Results:**

We present our findings under three inter-related domains. First, we describe what followed the clinical feedback implementation. Second, we present the context experienced as being complex and high on work-pressure. Third, we describe the situated rules about the priority between competing tasks.

**Conclusions:**

The preliminary results complement and contextualize understandings of known barriers to implementing ROM/CFS in clinical settings. We apply a socio-material perspective to discuss clinicians’ responses to complexity, implementation, and why some incentivized tasks prevailed over others regardless of therapists’ perceived benefits.

## Background

Research has demonstrated that clinical feedback systems/routine outcome measurement systems (CFS/ROM, hereafter called clinical feedback) can have positive effects on psychotherapy outcomes in mental-health settings. Clinical feedback involves systematically collecting client process and progress self-reports on standardized measures just before or after sessions, where both patients and therapist review the data to evaluate and re-evaluate the treatment plan [1]. Using clinical feedback may prevent treatment failure, reduce suicidality rates, and help patients who are not on-track, compared to treatment as usual [2–6]. Some governments have translated the benefits of clinical feedback into policies to improve mental-health services. Australia and New Zealand have collected outcome data for several years [7], and the United Kingdom’s Improving Access to Psychological Therapies (IAPT) program includes outcome monitoring and, in part, clinical feedback [8], and the Norwegian government has implemented clinical feedback as a part of standardized mental-health and substance-abuse treatment pathways [9].

Despite research evidence that demonstrates the potential effects of clinical feedback, which have been reified in clinical practice recommendations, studies indicate that few clinicians make use of clinical feedback even when they report having positive attitudes about it [10, 11]. Research reports and experiential accounts have identified barriers on both the individual and organizational level, such as philosophical issues, practical issues, time-consuming clinical-feedback tools, financial concerns, privacy and ethical questions, interference with autonomy, fear, and mistrust [12]. Boyce, Browne, and Greenhalgh’s [13] systematic review of implementation studies on Patient Reported Outcome Measures (PROM) found the salient issues were practical considerations, attitudes, and the perceived lack of causality between PROM use and improved patient outcomes.

Grounded in the evidenced-based medicine paradigm, Implementation Science (IS) attempts to bridge the gap between research evidence and routine practice in healthcare settings. It does so by identifying barriers and facilitators associated with the use of evidence in clinical practice, and evaluating strategies, theories, and models aimed at enhancing evidence-based practice [14–16]. Clinical feedback is associated with the Practice-Oriented Research’s (POR) integration of science and practice, which involves a broadening of the EBM-paradigm to also including complementary practice-based evidence. Through simultaneously serving clinicians, service delivery, and research, it takes a bottom-up perspective to enhancing therapy outcomes [17, 18]. Both approaches attend to questions about how to overcome implementation barriers, and clinical feedback researchers have adopted IS methods and techniques [19–21]. In addition, Boswell et al. [12] propose research on different implementation stages, single factors, and implementation models.

However, there is a lack of evidence to support the effectiveness of different implementation strategies and models [22, 23]. Multifaceted approaches and tailored intervention strategies have gained some support in the research literature [24, 25], and some evidence supports combining different intervention strategies with opinion leaders’ input to promote evidence-based practice in hospitals and primary care settings [26].

Some implementation challenges may be rooted in medical and health research, criticized for applying linear models that are more commonly used in industry and the natural sciences [27, 28]. For example, Greenhalgh et al. [29] argue that the sheer volume of implemented clinical guidelines produce complexity that, paradoxically, hinders further implementation. Braithwaite et al. [30] claimed that former implementation models based on a linear and reductionist paradigm failed to account for interactions and contextual and contingent features in the settings in which they took place, thereby masking the complexity of the practices. They have shown how the relationships between the components of complex adaptive systems (CAS),^1^ such as healthcare systems, are more important than their individual parts. In theory building, emergent patterns, factors, feedback loops, and both intended and unintended consequences, are integral for understanding implementation processes [30].

Modern public-service institutions are, to some extent, characterized by reconfigured professionalism [31], managerialism through systems of monitoring and control in healthcare institutions [32–34], commodification, financial and performance pressure [35], and high professional burnout due to increased workload [36]. Given these characteristics, it is pertinent to produce and implement knowledge that fits these complex settings, balances professional autonomy and managerialism, and integrates perspectives about how clinicians navigate between competing demands.

In this article, we apply a socio-material perspective to study complexity [37]. A socio-material perspective contrasts traditional views about agency [38–40]. Both within implementation science and within complex systems thinking, agency is limited to individuals, which can underestimate the inherent potential agency of implemented artefacts and standards, such as a clinical guideline or a clinical feedback system. In this perspective, context is constantly produced and re-produced by agentic relations. These relations operate within networks or arrangements in which the wholes’ capacity is not reducible to its individual components [39]. Groups or arrangements, and even institutions are considered temporary, and they are only stabilized by maintaining the relations that keep the arrangements together [38, 40].

These premises mandate research to examine how clinicians describe the arrangements that make up their work context, and how these form conditions that affect clinicians’ work and their use of clinical feedback. Knowledge produced from such research can provide new insights into implementation processes. Clinical feedback in complex public healthcare systems competes with multitudes of existing standards, routines, tools, and people that demand the clinicians’ attention. If one accepts these premises, clinical feedback system developers have limited power to determine the universal meaning of clinical feedback when it is translated to different settings and situations. The clinicians’ own interests, micro-politics inherent in standards, and other contextual factors will affect the practice of clinical feedback similar to other standards implemented for quality purposes [41]. In the current study, we (1) explore how clinicians describe everyday work, (2) explore how the implementation of a dynamic clinical feedback system, which was developed in-house, affects its setting, and (3) how points 1 and 2 affect clinicians’ decision-making and practice of clinical feedback.

## Methods

### Setting and context

The implementation object was Norse Feedback (NF), an in-house innovation of a mental health clinic in a Norwegian publicly funded district general hospital. NF’s purpose is to increase patient involvement and to support empowerment and service improvements through the systematic use of outcome and process documentation based on clients’ self-report data [42].

NF incorporates several basic features of similar systems, such as routinely measuring client progress and collecting feedback about treatment responses [43–45]. Moreover, the system is a computer-only adaptive system, which means that questions asked are adapting to each patient’s profile session by session. Clinicians and clients were participants in the development and subsequent improvements of NF, influencing the system’s features, the purposes it serves, and the items included in it [42]. The NF system (1) invites patients to report information on a personalized digital form prior to each treatment session, (2) instantaneously compares their responses to questions to norm-databases, and (3) provides the patient and clinician with a customized visual report to inform treatment. It also provides opportunities for direct alliances and requires feedback from the patient to the therapist.

NF is situated within an overarching action-research program, including various research projects and the systematic collection of clinical experiences with use. To continuously develop NF in annual cycles, the results of this study and other parallel studies form the basis for improvements. Alongside the present study, a hospital employed project developer worked to implement NF, support staff, and handle technical issues. Especially technical issues are, and have been a significant part of the continuous development process.

### Case study design

The case study design is pertinent to explore complex implementation processes in naturalistic settings with its combination of data sources, sampling, and analysis techniques [46]. We defined and bounded our case to be coherent with the overall research questions, and limited it to the first two hospital units to implement NF. Interviews and field observations were conducted over a predefined ten-month period.

### Data collection

We collected policy documents, meeting summaries, and field documents, and conducted in-depth interviews with stakeholders. Patients, individual therapists, milieu therapists, super-users, and managers were recruited using purposive and convenience sampling. Patients and staff who had not used NF and had no assigned role in its implementation were excluded from the study.

### Participants and data sources

Table 1 provides an overview of the participants. We recruited 15 female and eight male employees. Eleven worked at site A, and 12 at site B. In sum, four unit leaders, 14 individual therapists, four milieu therapists, and one project leader contributed their experiences. Five male and seven female patients participated. Half of the patients received treatment from the inpatient unit, the other half from the outpatient units. The first author interviewed the participants based on a semi-structured, open-ended interview guide. Participants at site A began the NF system a year before our implementation study. Participants at site B started the systematic implementation of NF at the beginning of our project. We, therefore, limited the interviews at site A to one per participant. At site B, we did a follow-up interview with participating staff to be able to track developments during the implementation process. Follow-up interviews were done between 3 and 6 months after the first interview. The interviews had an average length of 50 min, ranging from 30 to 80 min. The first author, with the help of an assistant moderator, interviewed the outpatients in a focus-group setting. Although not an inclusion criterion, in general, participants had a positive attitude towards the NF implementation.

**Table 1:** Overview of the participants

Participants^a^	N	Age range	Gender (n)	Site	Unit	Additional information
Inpatients	6	20‒60	Women (4) Men (2)	A	IP	
Outpatients	6	20‒45	Women (3) Men (3)	A (4) B (2)	OP	Focus group interview
Individual psychotherapists	14	25‒60	Women (8) Men (6)	A (6) B (8)	IP (11) Op (4)	Some were patients from both units; some had dual roles as NF superusers and unit coordinators
Milieu therapists	4	45‒60	Women (4)	A (3) B (1)	OP	Three had dual roles as NF superusers and unit coordinators
Management	4	35‒65	Women (3) Men (1)	A (2) B (2)	IP, OP and AT	All were unit leaders
Project leader	1					Project leader worked across all units

*IP* inpatient unit, *OP* outpatient unit, *AT* addiction treatment unit

^a^Nine of the eleven employees at site A were interviewed twice

We conducted our field research in ten educational courses, five meetings with key stakeholders, and seven morning-report meetings in one inpatient unit. The hospitals’ administrations provided archival data, such as meeting summaries, presentations, and policy documents.

The principal investigator observed and recorded various meetings with management and clinicians and of training events lead by the project leader. The project leader was interviewed on four occasions to gain insight into the implementation process. Archival data were analyzed, but used primarily to provide a context for the interviews and field studies. In-depth interviews constituted the primary source of data when constructing categories, and field reports were the secondary source. The inductive process used in the data analyses yielded several categories related to the research questions.

The present article reports one of three major findings of the case study. Another article discusses which clients clinical feedback systems fit [47], and another article reports how patients and therapists experienced NF to support therapeutic processes [48].

### Data analysis

We wanted to better understand implementation challenges in complex public health-care systems. The main empirical basis for exploring our research questions are located in interviews and field observations of employees. However, we also found it suitable to the context to include a few patient perspectives in the result section, where these adds nuances or complements employees’ perspectives. The total amount of data consisted of 37 h of interviews and 23 short field reports. The first author transcribed half the interviews and a professional transcription agency transcribed the other half.

We used QSR International’s NVivo software to structure the data material, and as a tool in the analysis process, in accordance with case-study methodology [46]. Case-study methodology does not favor specific data analysis methods. We found the stepwise deductive inductive methods’ (SDI) coding techniques were suitable for the analyses. SDI shares similarities with grounded theory, but replaces theoretical sampling with iterative tests for code development. Coding techniques resembles *open coding* or *eclectic coding* [49]. We coded the data in vivo, and merged, sorted, and reused codes during the process to keep the number of codes on a comprehensive level. We, then, grouped the codes based on thematic similarities before we developed concepts and categories. This last step was based on abductive reasoning, thematic similarities, and the frequency of sources coded.

The principal researcher’s academic supervisors audited the research process, read the data material, and reviewed the analysis for consistency and rigor.

## Results

**Fig. 1:**
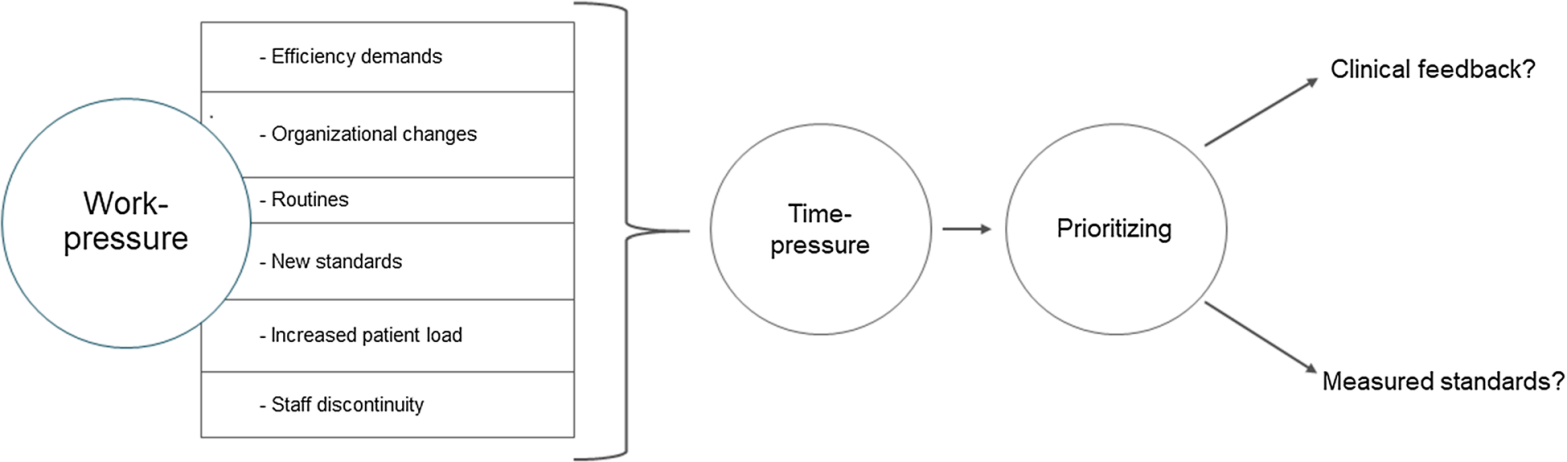
Making prioritizations in the narrow space between sessions

We present a brief illustration of our findings below. The first section covers issues that followed the clinical feedback implementation. The second section presents contextual issues that affected implementation, and the third section describes the situated and informal rules clinicians applied to manage competing demands in a complex setting (Fig. 1).

### What follows clinical feedback implementation?

Despite emergent impeding issues during the implementation process, the participants’ generally expressed being in favor of the implementation initiative. Still, all the issues that followed the implementation spurred reluctances and critical judgements. We address these issues under the subsequent themes.

#### Technical solution causes confusion and frustration at first

NF was a pilot project on a larger digital platform aimed at digitalizing common clinical forms and measures within the regional health trust—a parallel implementation in itself that transpired independently. The two separate implementation processes were a source of confusion and frustration at first. There was confusion because employees mixed the names of the projects, which on one occasion caused some staff to attend to the wrong introductory course. There was frustration because the serving digital platform had technical implementation challenges, which in turn, affected the user experience of NF. The problems included access difficulties, logistics, navigating the system, and many unexplainable errors. The opinion of the clinicians was, especially in the early phase of implementation, that when problems arose, it was critical to allocate sufficient resources to support functions. This need was particularly felt at the site that was not where the project’s management was located. In their view, physical presence could inspire and would signal determination. Clinicians found help was more accessible after a project developer was appointed to lead the implementation process further. A common issue was that the security and privacy protection policy required bank issued electronic IDs to access the clinical feedback system. Clinicians reported that many patients forgot to bring their ID and some did not have an electronic ID—for example, the elderly. Some patients were also under custody and, therefore, deprived of access to their bank account.

#### Logistical challenges

The logistics were set-up so that patients could pick up tablets at the reception office and answer the NF questions in the waiting room a few minutes prior to their session. This routine had different consequences. According to employees, patients forgot to come early, forgot to report to reception, or forgot to bring their E-ID. Clinicians’ said that office personnel sometimes forgot to offer clinical feedback to patients. When they did ask, patients sometimes had not heard about the clinical feedback system, which left the office personnel insecure. Some clinicians who were not located near the reception office had to administer the logistics themselves, which sometimes interfered with sessions. Due to these challenges, many clinicians told their patients to fill-out the measures at home the day prior to consultation. They wrote down their patients next scheduled session on a piece of reprinted paper with the NF’s URL-link, reminding their patients to remember to attend the session.

Since this was a hospital setting, it was also discussed whether the tablets were subject to the hygienic standards and needed sterilization between use.

#### Navigating and managing the system

Many clinicians found it difficult to schedule and look-up feedback reports because of difficulty with the interface and the terminology used on the serving platform—that is, the other active implementation that was occurring in parallel. Some felt navigation was illogical and counter-intuitive, and the terminology was misleading and not adapted to the clinicians’ language. A confusing example of both was the discovery of finding unanswered patient reports. These “unanswered forms” were filed under “finished mappings.” Later, as part of development of NF, feedback reports were automatically transferred to the medical health record, which made access easier since the therapists regularly consulted the health record before a session. One therapist regarded this integration as especially positive since “everything that just flows without the need for keystrokes is positive.”

#### Ethical and privacy concerns

Employees reported privacy and ethical concerns about both practical and principled issues, from introducing and completing the clinical feedback report to storing and retrieving feedback reports. Some clinicians worried about how the patients would feel about filling out clinical feedback forms in the waiting room, for example, how patients with paranoia would feel, or just if the tablet signaled that you were a patient. Outpatients we interviewed said they felt they were taken seriously when they were allowed to tell how they felt and thought through the feedback system. However, some of the interviewed inpatients felt that they did not get enough information, and that they complied without really knowing what they had consented to. This was often the case when temporary staff handed out the tablets on the scheduled day. Interviewed patients told that they often felt that temporary staff did not have knowledge about what they were handing out.

Many in management, as well as the clinicians, had concerns about unread feedback reports. They regarded it as disrespectful to the patients who spent time and effort to fill them out, and unethical to collect so much information without using it. There was also a fear that if a patient committed suicide, an unread feedback report could reveal an increased risk of suicide.

The health record integration of feedback reports made access a lot easier. However, the integration made the reports pre-approved in the journal system. This meant that the feedback report was not visible on a clinician’s work-list. One therapist said she discovered such a “hidden” report with a heightened risk score, and summoned the patient, only to find out it was a false high. Clinicians conveyed mixed feelings concerning medical health-record integration. They had patients who worried about others’ access to their reports, feeling that this was a private matter concerning only patients and their therapists. Others disagreed, arguing that the journal system was the most secure and safest way to store the feedback reports.

#### Motivation

After solving most initial technological and logistic challenges, super-users and leaders still reported that too few patients and therapists used the system compared to the organization’s expectations: “Now, the technical solution works. One cannot, in a way, put the blame on that anymore.”

The project management focused on unit leader ownership from early on. The need to continually motivate staff was a common understanding. They all regarded troublesome implementation as something normal. Unit leaders hoped to maintain the clinicians’ attention by talking about the upside when implemented, calming clinicians, keeping the discussions on a professional level, and appealing to the clinicians’ professional curiosity.

“That’s the problem [prioritization], because it competes with many other important activities—it does, but by focusing on advantages that we get out of clinical feedback, we will overcome the start-up problems quite efficiently.”

The project management and the unit leaders were reluctant to offer incentives to speed up the adaptation process. Their policy stated that clinical feedback should be the clinicians’ and patients’ tool to strengthen therapy. They feared incentives and measuring clinicians’ fidelity would only lead to reduced motivation. Clinicians did not feel pressure from the management to use NF. They felt the driving force was more their own motivation and sense of duty: “It has been said that everybody will use it, but then, it is just like it has become voluntarily after all.”

### Context: organizational changes and multiple demands increase work-pressure

Implementing clinical feedback, as we have seen, also raised a range of different issues other than those pertaining directly to the feedback system. These issues added weight to clinicians’ workload and became challenging also due to specific contextual characteristics that we attend to in this section. We further explore the setting that clinical feedback entered. We explored this by analyzing how employees perceived their everyday working conditions as a seed-bed for implementation.

Work-pressure sums up participants’ experiences of the organization as being in a constant flux caused by efficiency demands and a variety of concurrent activities that employees experienced as disproportional to the time allotted. More specifically, clinicians ascribed the causes behind work-pressure to different recurrent factors, such as efficiency demands, organizational changes, discontinuity among staff members, everyday routines, new standards, and increased patient load. Despite having a positive attitude towards clinical feedback, for many it became yet another thing to do among these competing demands. We explore these issues further in the following section.

#### Patient through-put demands

The therapists’ nearest leaders concurred with their experience regarding workload and pressure. They pointed out that increased patients referrals resulted in increased demand on patient through-put. “People come to me and complain about too much work and say ‘Can I get an exemption from receiving new patients?’ Allocating patients is an everlasting toil, and I hear that when I talk to… after all, I have regular meetings with other section leaders, or when you talk to people from other places in the country—it is like that. We struggle to get enough patients out so that we can take more in.”

#### Staff turnover

Turnover rates and discontinuity made implementation challenging and vulnerable. Many therapists pointed to high turnover among staff and key personnel as demanding. Continuity was especially lacking among specialist psychologists and psychiatrists. “Many temporary psychiatrists come and only stay here for 1 week, 2 weeks, a month.” Participants stated that temporary employees did not have time to acquaint themselves with NF. Additionally, when there was a staff shortage, each therapist had more patients, more work, and less time to engage with NF, or they simply forgot. Due to the staffing situation, some patients had to change therapists. When this happened, NF reports sometimes were still connected to former therapists. Staff also reported about turnover and sick leaves among the leadership that affected the processes’ continuity.

#### Competing demands

Most clinicians described the extra-therapeutic work as extensive. One leader said: “There are extremely many demands on what we should do.” Similarly strong expressions were common to describe everything clinicians needed to relate to besides NF. Topics revolved around registration demands, deadlines, and tasks in connection with patient intake. One clinician said that even if the task itself was small; the sum of it became large. Some clinicians described the situation as a never-ending flow of other things that happened: “There are new forms to adhere to all the time,” and “We have systems for everything, and they all come with their own data tool, with their own login.” One senior clinician said the things they were asked to be involved in and to comment on were “endless and insatiable.” Another senior clinician said that years of this situation, perhaps, made her blunt to new things.

Many felt that regular tasks and demands were not clinically relevant, but still prioritized: “One develops routines which suggest that every patient has the same needs.” In meetings with management, one therapist said that the agenda seldom was about professional issues. It was rather about routines, and administrative and economic issues. One example of tasks mentioned by some clinicians that they felt were irrelevant, was the newly implemented national suicide screening. It demanded actions, but drew attention away from significant clinical work.

“You know, it is very intensive, and it is obvious, all these new things become hard to swallow, because you do not feel the same needs as the management. Because, they want to make sure we have good routines, while the individual therapists say that we do not have time to follow all the routines on the dot. We have to be pragmatic, because this is how ordinary work days are.

#### Behind on work

Workloads made many feel overwhelmed and created a feeling of never doing a proper job. All tasks had to be done between sessions: “Actually, it is like if you have many things to do already, you now get another thing. It is not as if something is removed, or made easier. It is just more work, and more to relate to.” They did some tasks just once, others on a more regular basis, and some were not scheduled to be part of any routine. For some fresh therapists, the workload meant lack of time for professional development, which meant more work brought back home.

### The situated rules of priority: counted work counts

#### Clinical feedback disappears in the crowd

Although all interviewed clinicians were positive about implementing clinical feedback, their competing demands, expectations, and personal situation reduced their use of clinical feedback. Several clinicians described lack of time, due to work-pressure, as a challenge both to learning NF and to practicing it. They experienced that clinical feedback was “drowned” in other tasks. It was “just one of these things that slides away,” and that “there are so many projects, and so many new things. Everything is at the expense of everything.” A few therapists had their 1st year of practice, and felt that differentiating between expectations was challenging. “There is something new all the time, so in a way, it [NF] was just a part of it all.”

However, one therapist expressed the view that even though clinical feedback took time it would probably save time in the end, especially considering the time required to develop a therapist-patient relationship. Especially outpatients supported this view. Some of them felt that using clinical feedback saved time during the initial talk about how things were, and that it speeded up the process of presenting their stories. Another therapist who was initially reluctant to integrate another tool in the dialectical behavioral therapy process, found the diary card became richer after patients began answering the clinical feedback.

#### Signs of voluntary tasks

Participants who were clinicians did not recall any new tasks that were later eliminated, but they said tasks could fade away. If management did not follow-up on tasks in any formal way, some clinicians would regard it as a sign of voluntariness. One therapist said she always worried what would happen when she forgot, or did not follow-up on new tasks, and that this worrying could last for a very long time. Another therapist reported that implementations survived only if clinicians quickly regarded it as a good tool that saved time. If the management no longer had their eyes on implementation, then a task only survived as long as the clinicians regarded it as a meaningful thing to do.

Clinicians said they had strategies for how to prioritize multiple demands in a limited amount of time. Tasks regarded as useful could sometimes be given priority, but more often urgent tasks were prioritized, for example writing patient referrals. National guidelines and tasks regulated by law were given high priority among clinicians. Incentives often followed these tasks, such as counting or reminders—computer-generated, or by email. All statutory tasks came before NF. Clinicians felt that the management more closely followed up tasks initiated from “the top.” By “the top” they meant the ministry of health, directorate of health, or the regional health trust administration. Even if management informed clinicians of clinical feedback’s mandatory status, no consequences were enacted for avoiding to inform patients, or if feedback reports were not followed-up. “I am not afraid of being hanged by the Chief County Medical Officer because I did not start up NF.” Not “counting” or imposing incentives to increase use was a deliberate choice of the steering group, fearing it would be associated with measured tasks that felt irrelevant, and created resistance. Many clinicians supported this view, but one therapist, although skeptical of the “counting regime,” thought since counting is a part of everyday life it could perhaps help her to practice clinical feedback:

“We are measured on everything. Referral rates and absolutely everything, so why then can one not just measure if therapists use NF? For example, I am prepared to be questioned: ‘Why have you only eight out of twenty?’ I think it is an interesting question. Perhaps I need help to get 12 out of twenty.”

## Discussion

### Interconnected barriers

The staff, even though they had a positive attitude towards clinical feedback, had to negotiate the competing demands of implementing a comprehensive clinical feedback system and their routine work in a setting characterized by high workloads and time-pressure. We showed how and why some tasks prevailed over clinical feedback. We have also described multiple heterogeneous standards that worked performatively together in some socio-material arrangements, but became internally disruptive in others, especially when turned into micro-political means. In the following, we discuss why this might happen. To this end, we apply a socio-material perspective, introduced by Timmermans and Berg [37], to discuss responses to complexity, with special attention to what embedded micro-politics do, as part of both creating complexity and guiding therapists’ actions towards intended goals. Implementing clinical feedback can be regarded as the implementations of different standards in one wrapper that need to be compatible with other standards at work to succeed; for example, technical standards, therapeutic standards, or validity standards [37, 41]. During the implementation phase, when NF was in the process of achieving its intended purpose, it also became a frustration and work-producing machine due to the extended workload it created. The system was associated with many diverse and not always understandable technical challenges, resonating with known practical and philosophical issues discussed in the implementation literature [12]. While the NF project management solved many problems, additional concerns and relationships that needed persistent problem-solving emerged. The complex and unpredictable nature of health-care systems points to the importance of anchoring clinical feedback in an action-research program that addresses continual improvement efforts.

The feedback system was not equipped with sufficient conditions for a smooth integration, nor was the social processes of adapting it sufficient. It came with logistic, managerial, privacy, and technical challenges, and thus, became disruptive and re-configurative. Clinical feedback proved difficult to fit within this complex system during this phase.

### Unintended chains of events

Considering this case through the lens of complex adaptive systems (CAS) theory, idiosyncratic events generated new patterns and influenced chain of events [30]. For example, hospital security standards led to the need for patients to bring a Bank ID to the hospital. In turn, this led to the need to remind patients, presupposing the ability and resources of staff to implement reminder routines. Moreover, standard letters needed to be reformulated to include reminders in written communication in such a way that they were understood, and automated letters with meeting times drawn from the scheduling system needed to be changed to allow sufficient time in the waiting area before sessions. However, such letters were standardized and regional, and changing them involved work tasks for workers initially far outside the implementation scope. This example illustrates CAS responses when trying to solve issues, and how one issue might bite the next one in its tail. Considered within a relational ontology, this illustration may improve our understanding of previously reported barriers to clinical feedback. For example, Boswell et al. [12] reported that technical issues are one key barrier. As illustrated, a technical issue, understood contextually in a CAS framework, evokes relationships beyond the technical realm, reorganizes work-flow outside the technical scope, and thus, increases the barriers to implementing clinical feedback [12, 50, 51]. As another example, for a limited time the suicidal screening item of the clinical feedback tool was too sensitive, resulting in false highs. At the same time clinical feedback reports became automatically registered in the electronic patient journal as read and approved, which also meant that it was not visible on the list of the clinicians’ daily work tasks. This made clinicians concerned about the consequences of both real highs and false highs that were not discovered. Additionally, they became worried about how a governmental audit would assess the legal status of the clinical feedback reports, fearing sanctions.

### Outcomes other than those intended

The results also underscore how the apparent redundancies that follow implementations require attention. Cumbersome navigating and managing tablet logistics were small issues in themselves, but when combined with other pressing issues they added up to be significant concerns. As evident in the participants’ experiences, the pressured work setting did not have space for redundancies. Concretely, participants described an ever-changing environment of staff turnover, efficiency demands, and competing tasks that, in sum, produced work-pressure. Within this environment, although participants expressed their acceptance that NF represented good intentions and was an initiative to improve service quality, the situated meaning of the clinical feedback was that it increased the work of clinicians. From the perspective of some of the clinicians, many of the existing standards did not add up and build capacity to help them perform better within a therapist-patient arrangement.

### Re-configurative sanctions

The standards of micro-political efficiency were inherent sanctions. When having to choose between competing demands, situated priority rules emerged among the staff. Counting meant a lot, statutory tasks and national guidelines had priority, and the higher in hierarchy that the standards originated, the higher the fidelity was. It was a pretended bureaucratic exercise, a strategic balance between performing and reporting [35].

Prioritizing was especially strong among the work demands in the often narrow space between sessions that had consequences for clinical feedback. Leaders and the steering committee were reluctant to count and incentivize NF. It was intended to be a tool to enhance therapy, and not to be associated with negative administrative work. The main strategy to improve NF uptake was motivational work, training, constant problem-solving, and embedding feedback reports as part of clinical meetings. Counter to this strategy, the work of other incentivized standards seemed more efficient in affecting therapists’ behavior in between sessions. Competing standards worked as micro-political agents of socially inscribed intentions, and this meant that they reconfigured how therapists acted, away from desired therapist-patient related work, towards wanted actions decided by distant actors. These findings show competing quality improvements logics and policies, and support critical perspectives on public health-care governance [33, 52].

However, clinicians did not regard all standards as disruptive. When clinical feedback, for example, eased their everyday work, adherence would increase regardless of incentives, and the integration of feedback reports with medical records was a step in that direction. In this workplace where therapists were under constant pressure, the use of clinical feedback rested solely on the therapist’s conscience and interests, and not following-up NF had no consequences. As such, the micro-political governance in this case seems incongruent. On one hand, incentives can be effective tools in regulating clinicians’ behavior, and simultaneously, they can be necessary because of the ever-changing working environment and work-pressure they partake in producing. On the other hand, clinicians accept changes that feel relevant within their performative arrangements, but clinical feedback without incentives risks being given less priority due to prioritization of tasks that are less performative, but more incentivized.

### Implications for implementation of clinical feedback in practice

Implemented in complex adaptive healthcare systems, incentivized standards produced by government actors have a fast lane to frontline professionals treating patients. Therefore, the normative question becomes: given the chain of outcomes other than the ones intended, should it not also be the implementer’s obligation to attend to all aspect of their product? Is not the redundant part an outcome, as well as the intended one? One risk of not accounting for complexity is that redundant parts are privatized as being the responsibility of actors in the CAS. Moreover, if redundancies outweigh intended outcomes, how can we still stick to essential definitions of the implemented object? We showed in another study how NF as an idiographic clinical feedback system goes into a performative therapist-patient arrangement [48]. Thus, the coined questions above are vital for CAS implementation of clinical feedback.

The NF is a digital solution only that relies on, and needs to be compatible with, existing technological infrastructure. This, and that its implementation demands considerable human and technological resources, limits its scope of implementation to health systems with less resources.

### Implications for implementation research on clinical feedback systems

Our findings challenge an essential and linear understanding of clinical feedback implementation, and problematize how other outcomes than the intended ones cannot be treated as barriers in the hands of the adopters to solve. A contrary approach, the socio-material perspective, shows that what is redundant is part of a continuum of events produced interacting with the setting.

We have argued that redundancies and other-than-outcomes could be beneficially integrated into implementation science models, as suggested by Braithwaite et al. [30]. Finally, we have exemplified how CAS can provide a useful framework for understanding implementation in complex health organizations.

## Data Availability

The datasets generated during the current study are not publicly available due to individual privacy considerations.

## Footnotes

1 CAS is defined as “a dynamic, self-similar collectivity of interacting, adaptive agents and their artefacts” [30].
